# Increased Proviral DNA in Circulating Cells Correlates with Plasma Viral Rebound in Simian Immunodeficiency Virus-Infected Rhesus Macaques after Antiretroviral Therapy Interruption

**DOI:** 10.1128/JVI.02064-20

**Published:** 2021-02-24

**Authors:** Widade Ziani, Jiasheng Shao, Xiaolei Wang, Kasi Russell-Lodrigue, Yao-Zhong Liu, Luis J. Montaner, Ronald S. Veazey, Huanbin Xu

**Affiliations:** a Tulane National Primate Research Center, Tulane University School of Medicine, Covington, Louisiana, USA; b Department of Biostatistics and Data Science, Tulane University School of Public Health and Tropical Medicine, New Orleans, Louisiana, USA; c The Wistar Institute, Philadelphia, Pennsylvania, USA; Ulm University Medical Center

**Keywords:** SIV/HIV, viral reservoir, cell-associated viruses, antiretroviral therapy, proviral DNA and viral rebound, proviral DNA, viral rebound

## Abstract

The human immunodeficiency virus (HIV) reservoir is responsible for persistent viral infection, and a small number of mosaic latent cellular reservoirs promote viral rebound upon antiretroviral therapy interruption, which is the major obstacle to a cure. However, markers that determine effective therapy and viral rebound posttreatment interruption remain unclear. In this study, we comprehensively and longitudinally tracked dynamic decay of cell-associated viral RNA/DNA in systemic and lymphoid tissues in simian immunodeficiency virus (SIV)-infected rhesus macaques on prolonged combined antiretroviral therapy (cART) and evaluated predictors of viral rebound after treatment cessation. The results showed that suppressive ART substantially reduced plasma SIV RNA, cell-associated unspliced, and multiply spliced SIV RNA to undetectable levels, yet viral DNA remained detectable in systemic tissues and lymphoid compartments throughout cART. Intriguingly, a rapid increase of integrated proviral DNA in peripheral mononuclear cells was detected once treatment was withdrawn, accompanied by the emergence of detectable plasma viral load. Notably, the increase of peripheral proviral DNA after treatment interruption correlated with the emergence and degree of viral rebound. These findings suggest that measuring total viral DNA in SIV infection may be a relatively simple surrogate marker of reservoir size and may predict viral rebound after treatment interruption and inform treatment strategies.

**IMPORTANCE** Viral reservoirs are involved in persistent HIV infection, and a small number of mosaic latent cellular reservoirs promote viral rebound upon analytical treatment interruption, which is the major obstacle to a cure. However, early indicators that can predict resurgence of viremia after treatment interruption may aid treatment decisions in people living with HIV. Utilizing the rhesus macaque model, we demonstrated that increased proviral DNA in peripheral cells after treatment interruption, rather than levels of proviral DNA, was a useful marker to predict the emergence and degree of viral rebound after treatment interruption, providing a rapid approach for monitoring HIV rebound and informing decisions.

## Abstract (precis)

Viral reservoirs are involved in persistent HIV infection, and a small number of mosaic latent cellular reservoirs promote viral rebound upon analytical treatment interruption, which is the major obstacle to a cure. However, early indicators that can predict resurgence of viremia after treatment interruption may aid treatment decisions in people living with HIV.

## INTRODUCTION

Human immunodeficiency virus (HIV) establishes latent reservoirs extremely early after infection, yet combination antiretroviral therapy (cART) fails to eliminate latently infected cells, despite preventing viral replication, allowing the virus to persist throughout the lifetime (1–4). Early cART initiation significantly reduces viral reservoir size, yet viral DNA remains detectable in latently infected cells, leading to rebound viremia soon after treatment interruption (5–7). A variety of assays have been developed to measure viral reservoir size (unspliced [US] and spliced viral RNA transcripts, integrated proviral DNA, and intact or defective proviral DNA). However, comprehensive dynamics of viral decay in systemic and lymphoid tissues in animal models under prolonged cART and markers to predict viral rebound after treatment discontinuation remain unclear.

In the HIV/simian immunodeficiency virus (SIV) life cycle, the virus produces unspliced RNA (∼9 kb) and more than 100 differentially spliced transcripts (cell-associated viral RNA) in two class sizes (early [∼2-kb] and late [∼4-kb] RNA) (8, 9), which afford distinct information for viral replication, pathophysiology, and efficacy of antiretroviral therapy (10–12). The unspliced transcript is responsible for *gag*/*pol* translation and packaging of the viral RNA genome. The 4-kb incompletely spliced transcripts encode viral proteins Env, Vif, Vpr, and Vpu following export into the cytoplasm. The multiply spliced (MS) viral RNAs (∼2 kb) express *trans*-activation response element (Tat), Rev, and Nef. Of these, Tat protein drives high-level HIV/SIV transcription by TAR-binding transactivation, whereas Rev is necessary for efficient nuclear export of the various HIV/SIV RNAs that encode structural and regulatory viral proteins (13–16). All of these are necessary for the process of reverse transcription, proviral integration, transcription, viral protein expression, and virion assembly (17–21). Throughout the HIV/SIV life cycle, various viral nucleic acids, representing different infectious statuses and clinical significances (10, 11, 22–26), can be measured in tissues. Given HIV/SIV RNA/DNA forms in the viral life cycle, their existence and abundance might be helpful to estimate reservoir size and latency. (i) HIV/SIV full-length, unspliced RNA expresses Gag and Gag-Pol capsid proteins as well as genomic RNA for viral packaging; thus, HIV *gag* RNA transcripts represent bona fide HIV unspliced RNA involved in viral replication (27, 28). (ii) Correctly spliced HIV/SIV *tat*/*rev* RNA can express functional proteins, which are indispensable for viral replication and production (29). (iii) Episomal two-long-terminal-repeat (2-LTR) circles are extrachromosomal bystander products generated upon failed integration of HIV/SIV (26). (iv) Finally, a pool of integrated proviral DNAs, only ∼11.7% of which represent genetically intact HIV genomes in treated patients (30), are believed to be responsible for most viral rebound after ART cessation. Remaining defective proviruses (∼88.3%) may have the capacity to produce viral proteins, leading to host responses (31–33), but levels of integrated cell-associated intact viral RNA or DNA could be indicators to define and quantify productive and/or latent reservoirs. Integrated HIV proviral DNA is a fundamental constituent of the viral reservoir (4, 22, 24, 34–37), and its existence may provide a major resource to fuel viral rebound once treatment is withdrawn.

In this study, we utilized the SIV macaque model to mimic antiretroviral therapy in HIV infection and longitudinally monitored the decay of cell-associated SIV RNA/DNA in peripheral blood and lymph nodes collected from animals on cART (up to 20 months) to seek potential markers to predict viral relapse and viral rebound, which could inform human treatment strategies.

## RESULTS

### Dynamics of plasma viremia, peripheral CD4^+^ T cells, and cell-associated SIV RNA/DNA in SIV-infected rhesus macaques after 20 months of treatment and interruption.

**FIG 1:**
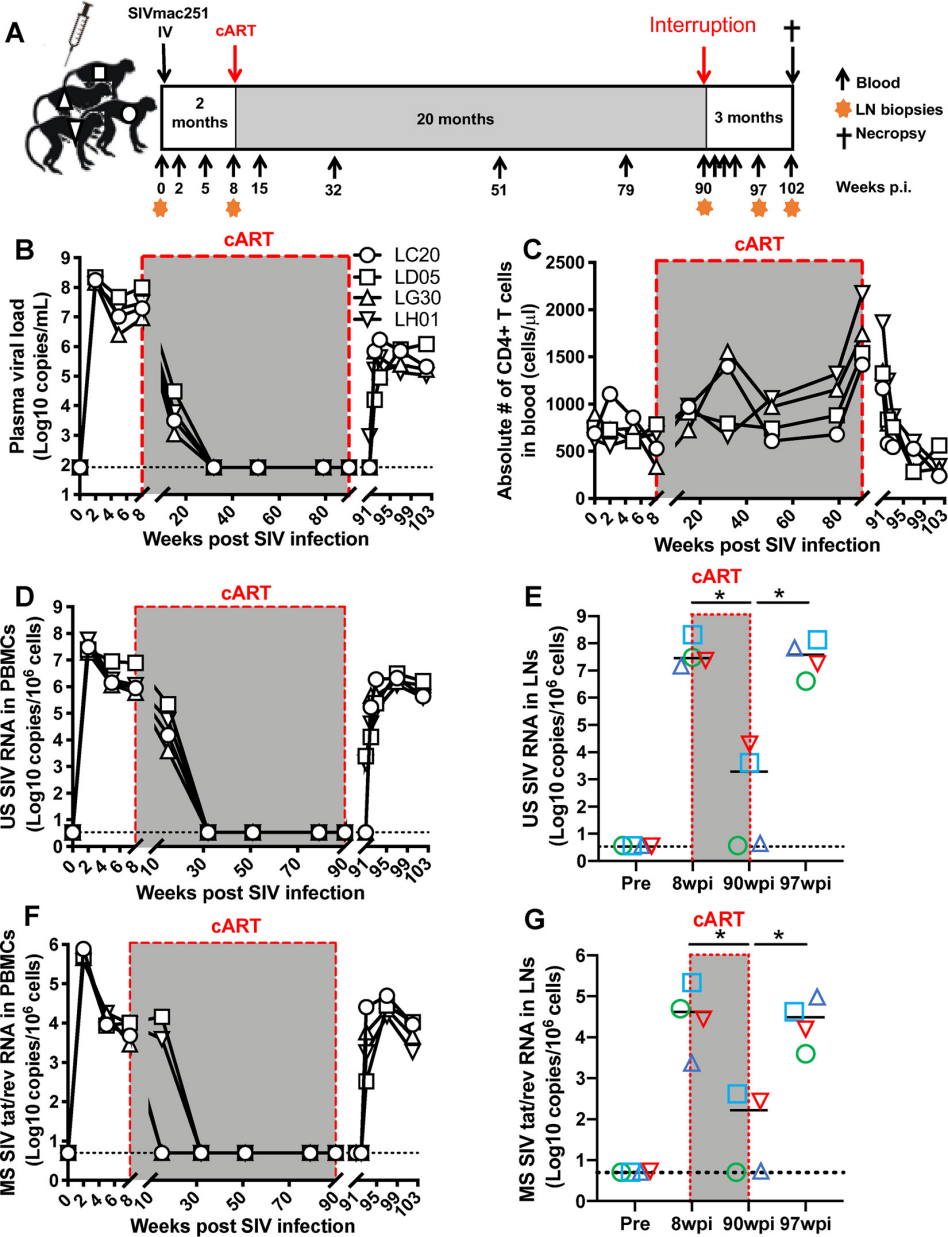
Dynamics of plasma viral load, peripheral CD4^+^ T cells, and cell-associated SIV RNA in PBMCs and lymph nodes of SIV-infected macaques on prolonged antiretroviral therapy. (A) Schematic representation of the study protocol and schedule for SIV inoculation and prolonged cART (up to 20 months) in rhesus macaques; (B) dynamics of plasma viral load in SIV-infected macaques on prolonged cART; (C) absolute numbers of peripheral CD4^+^ T cells in SIV-infected macaques on prolonged cART; (D) dynamics of cell-associated unspliced (US) SIV RNA in PBMCs from SIV-infected macaques on prolonged cART; (E) changes in cell-associated US SIV RNA in lymph nodes of SIV-infected macaques on prolonged cART; (F) dynamics of cell-associated multiply sliced (MS) SIV *tat*/*rev* RNA in PBMCs from SIV-infected macaques on prolonged cART; (G) changes in cell-associated MS SIV *tat*/*rev* RNA of lymph nodes of SIV-infected macaques on prolonged cART. The dotted line represents the limit of detection (LOD) calculated as described in Materials and Methods. The cell-associated US and MS SIV RNAs are expressed as copies per 1 million cells. *, *P < *0.01, determined by two-tailed paired *t* test. The designations LC20, LD05, LG30, and LH01 represent individual animals.

Adult macaques were intravenously inoculated with SIVmac251 and then given 20 months of combined antiretroviral therapy (cART) initiated 8 weeks after SIV inoculation. Blood and lymph node biopsy specimens were collected at scheduled time points (Fig. 1A). Plasma viral load and peripheral CD4^+^ T cells were longitudinally monitored. As indicated in Fig. 1B, plasma viremia peaked at 14 days postinoculation (dpi) and suppressive treatment significantly reduced viral loads to undetectable after 3 months of cART and afterward. Consistent with other reports (2, 38, 39), prolonged cART had a limited impact on viral clearance, as shown by a rapid viral rebound after treatment interruption. Accordingly, peripheral CD4^+^ T cells decreased after SIV infection, gradually recovered with ART treatment, yet rapidly declined following treatment cessation (Fig. 1C). These results showed that cART suppressed viral replication, but viral reservoirs were not eliminated even after 20 months of sustained treatment.

Cell-associated HIV RNA represents active viral transcription and is a biomarker of viral persistence, which is effectively suppressed by treatment yet also increases upon reactivation of HIV latency (19, 40). To track kinetics of cell-associated viral RNA in SIV-infected animals after cART, unspliced (US) and multiply spliced (MS) SIV RNAs were comprehensively measured longitudinally in both peripheral blood mononuclear cells (PBMCs) and lymph nodes of SIV-infected macaques. In parallel with the dynamics of plasma viral loads, both US and MS SIV RNAs were reduced to undetectable levels after cART and gradually increased after treatment interruption (Fig. 1D and F), trending with plasma viral loads. In contrast to the case with PBMCs, viral RNA was still detectable in lymph nodes of two animals (LD05 and LH01) even after 20 months of cART (Fig. 1E and G), suggesting that lymphoid tissues may have suboptimal anti-HIV drug levels and thus serve as sanctuary sites, resulting in persistent viral RNA transcription and fueling rapid viral rebound after treatment interruption (41–44). Thus, although cART may reduce plasma viral load and cell-associated SIV RNA, it may fail to fully suppress viral transcription in lymphoid tissues, possibly due to poor drug penetration in tissues (42).

### Levels of total viral DNA are significantly reduced, yet proviral DNA remains detectable in peripheral blood and lymph nodes throughout treatment.

**FIG 2:**
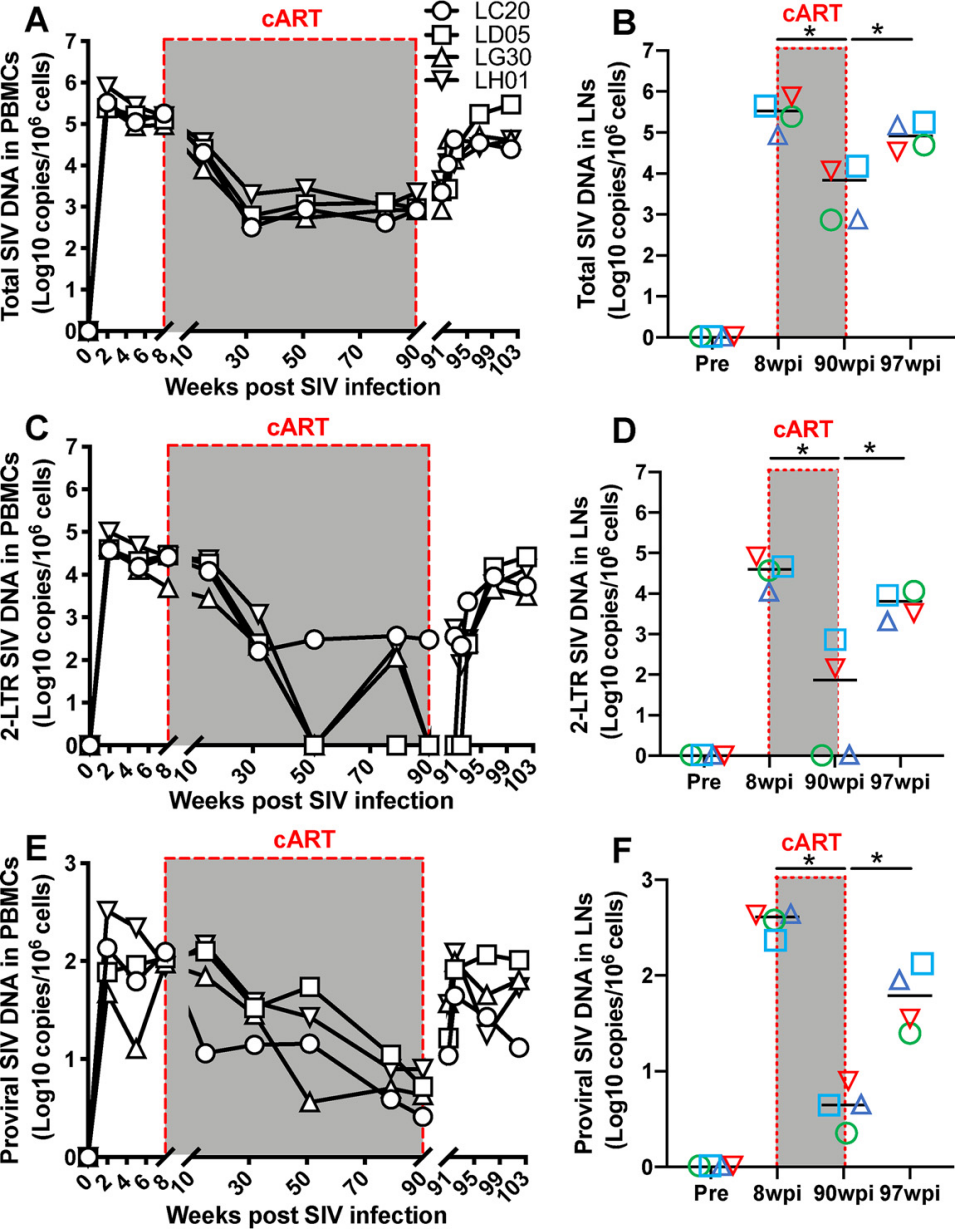
Dynamics of decay of cell-associated total, circular 2-LTR, and SIV DNAs in PBMCs and lymph nodes in SIV-infected macaques on prolonged antiretroviral therapy. (A and B) Changes in cell-associated total SIV DNA in PBMCs and lymph nodes from SIV-infected macaques on prolonged cART; (C and D) dynamics of cell-associated episomal circular SIV 2-LTR DNA in PBMCs and lymph nodes from SIV-infected macaques on prolonged cART; (E and F) decay of cell-associated integrated proviral DNA in PBMCs and lymph nodes from SIV-infected macaques on prolonged cART. Cell-associated SIV DNA is expressed as copies per 1 million cells. *, *P < *0.01, determined by two-tailed paired *t* test.

Persistence of HIV/SIV DNA, especially intact genomic proviral DNA, is believed to be the major barrier to a cure for HIV, as this is responsible for viral rebound after ART cessation (25, 45, 46). Proviral DNA is a unique marker to evaluate residual reservoirs when plasma viremia is undetectable in ART-treated patients (22, 34–36). In this study, viral DNAs, including total SIV DNA (all viral DNA forms targeting the LTR U5 region), 2-LTR DNA circles (circular SIV DNA targeting ligated junction of the ends of two LTRs), and proviral DNA (chromosomal *Alu*-based viral DNA only), were longitudinally measured in animals at different time points. At week 2 after SIV infection, levels of proviral DNA peaked, and cART treatment significantly reduced levels in both PBMCs and lymph nodes. Notably, total and integrated proviral DNAs remained detectable throughout treatment but rapidly increased within 2 weeks after treatment interruption, reaching consistently detectable levels after 20 weeks of cART (Fig. 2A and E). In contrast, plasma and PBMC-associated SIV RNA remained largely undetectable at these time points (Fig. 1D and F). “Blips” of peripheral 2-LTR DNA were detected in lymphoid tissues of three animals during treatment (Fig. 2C), suggesting that ongoing low-level SIV replication persisted despite undetectable viral RNA in plasma. However, the significance of sporadically detected 2-LTR is still unknown. Compared with detectable US and MS RNA levels in only two animals on treatment (Fig. 1E and G), proviral DNAs were consistently detectable in lymph nodes of all 4 animals examined (Fig. 2B, D, and F), demonstrating that proviral DNA was likely maintained at considerable levels in lymphoid tissues compared to peripheral blood.

### Peripheral proviral DNA decay under cART and its rapid increase after treatment interruption.

**FIG 3:**
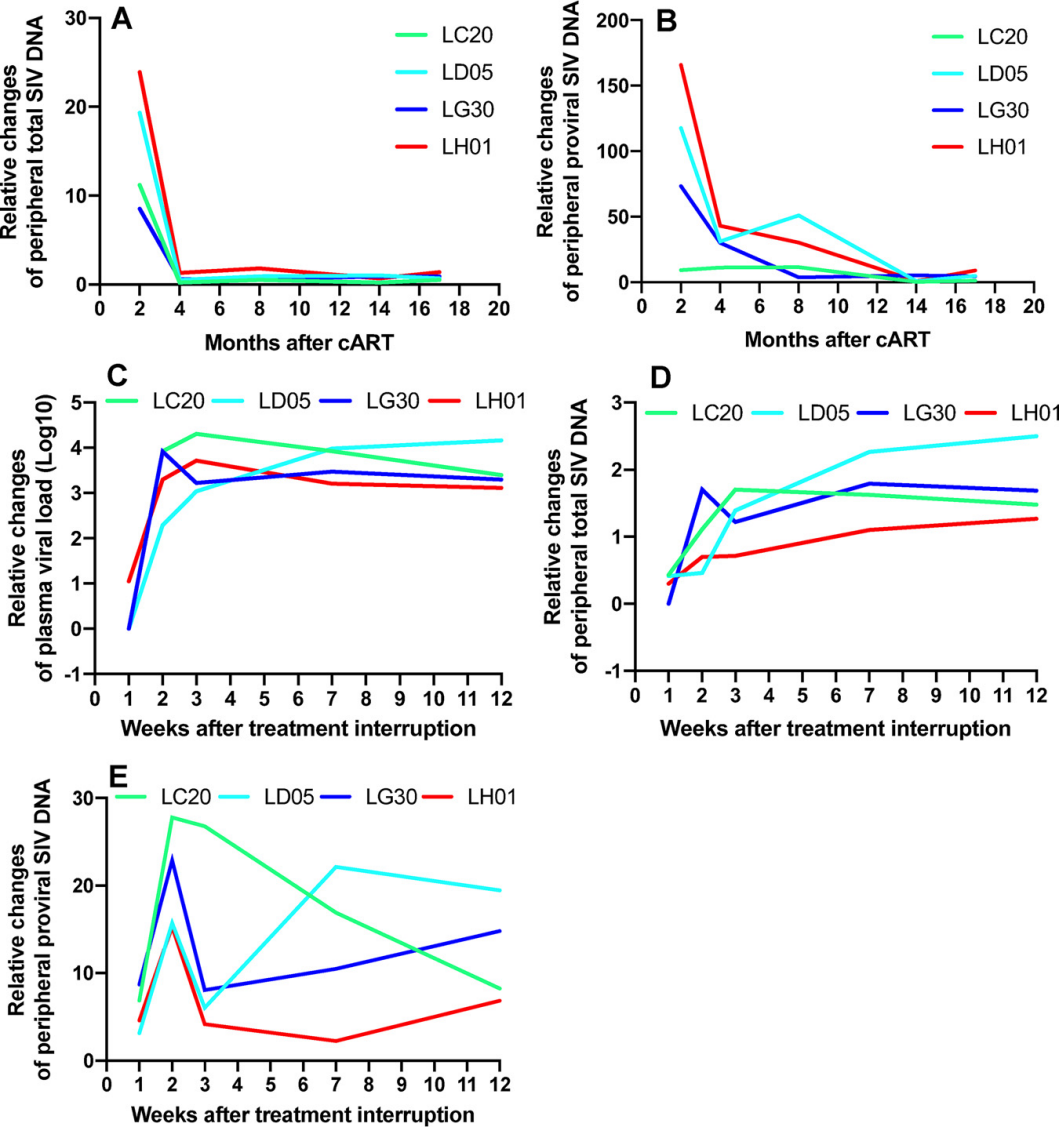
Viral decay under treatment intervention and rapid increase of viral DNA in PBMCs after antiretroviral therapy interruption. Shown are changes in ratios of total SIV DNA (A) and proviral SIV DNA (B) in PBMCs after treatment, compared with the baseline at the time point before ART initiation, and fold increase of plasma viral load (C), total SIV DNA (D), and proviral SIV DNA (E) in PBMCs after cART interruption, compared to baseline at the final time point of ART. Note that the data points between week 1 and 2 ATI for both LG30 and LC20 animals overlap (C). The levels of proviral SIV DNA significantly increased as early as 1 week after treatment interruption, when SIV RNA was essentially low or undetectable. Cell-associated SIV RNA/DNA are expressed as copies per 1 million cells.

Given absolute copies of viremia and SIV DNA in PBMCs throughout SIV infection (Fig. 1 and 2), we further calculated relative changes of both total and integrated proviral DNA under cART, expressed as the ratio of viral DNA under cART at different time points compared to levels at “baseline” before treatment initiation. Proviral DNA decreased after treatment (Fig. 3A and B). It has been reported that latently infected cells replenish reservoirs through clonal expansion despite ART, maintaining viral persistence (3, 47–49), yet our data suggested that viral DNA in blood cells did not increase under treatment when viremia was undetectable. To examine the effects of treatment interruption on viral rebound, we analyzed relative changes of proviral DNA in PBMCs after treatment cessation, especially in the critical early time period (5 to 21 days after treatment interruption), after which viral loads rebound in the majority of HIV/SIV-infected individuals after treatment interruption (5, 49). As indicated in Fig. 3D and E, SIV DNA, especially integrated proviral DNA, rapidly increased as early as week 1 and reached a peak at day 14 after treatment interruption, in concert with subsequent increases in plasma viral loads (Fig. 3C). These results suggest that the majority of the rapidly increasing proviral DNA detected is productive rather than defective. At ∼2 weeks after treatment interruption, viral transcription and replication were actively initiated, as indicated by detectable plasma RNA and cell-associated SIV RNA and 2-LTR DNA emerging in all animals examined (Fig.1 and 2). These findings suggest that a rapid increase in proviral DNA occurs after treatment interruption, likely resulting in plasma viral rebound.

### Increased proviral DNA after treatment interruption correlates with the emergence and degree of plasma viral rebound.

**FIG 4:**
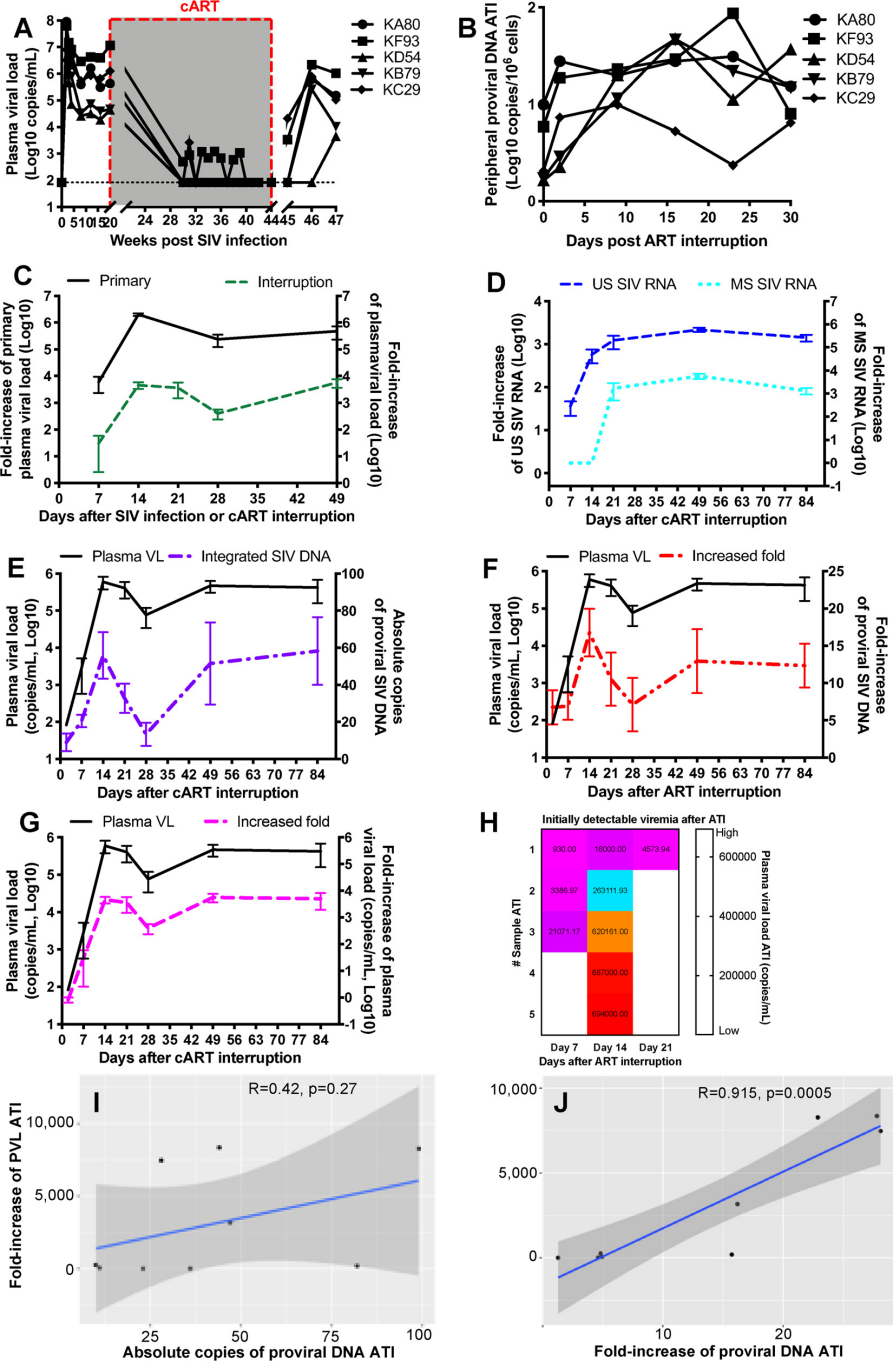
Correlations between proviral SIV DNA with plasma viral rebound after ART interruption. (A) Dynamics of plasma viral load in an additional 5 SIV-infected macaques on 6-month cART. (B) Dynamics of cell-associated integrated proviral DNA in PBMCs after treatment interruption. (C) Dynamics of plasma viral load after primary infection correlated with levels after treatment interruption. (D) Dynamics of plasma viral load and US and MS SIV RNAs in PBMCs after treatment interruption. (E and F) Dynamics of plasma viral load, absolute copies of proviral SIV DNA, and fold increase in proviral SIV DNA post-treatment interruption. (G) Dynamics of plasma viral load were in line with its fold increase after treatment interruption, compared with the plasma viral load at the final time point under ART. (H) Comparison of viremia and time to viral resurgence when the viral rebound was first detected in individual animals after treatment interruption. (I and J) Correlation of fold increase in plasma viral load (PVL) (degree of viral rebound) with absolute copies of integrated DNA (*R* = 0.42; *P* = 0.27) (I) or fold increase in proviral SIV DNA in PBMCs (*R* = 0.915; *P* = 0.0005) in 9 animals with analytic treatment interruption (ATI) (J). Shaded areas reflect 95% confidence intervals. The fold increase in proviral SIV DNA was determined by the ratio of integrated proviral DNA after and before treatment interruption.

To monitor changes in cell-associated SIV RNA/DNA and concurrent plasma viral RNA loads after treatment cessation, we included blood samples from 5 additional SIV-infected animals after 6-month treatment interruption. Following treatment interruption, the increase levels of both cellular US and MS SIV RNAs were essentially followed by similar increases in plasma viremia (Fig. 4B). Importantly, the dynamics of proviral DNA in PBMCs also correlated with plasma viral loads, as indicated by a rapid increase at day 7 and peak at day 14, followed by a drop and eventual set point (Fig. 4C and D). The dynamics of plasma viral load after treatment interruption was also similar to that in primary/acute SIV infection (Fig. 4A and E). It is controversial whether levels of proviral DNA after treatment interruption determine reservoir size, viral rebound, and time to recrudescence of virus (22, 34–36, 50, 51), as we assume that timing and extent of viral rebound after treatment interruption may affect treatment efficacy. Here, our data showed that levels of plasma viremia closely followed the extent of increased plasma viral load after treatment interruption (Fig. 4E). To further evaluate viral rebound biomarkers after treatment interruption, three parameters were analyzed for correlations: fold increase of proviral DNA, timing of initial rebound, and degree of viral rebound. For example, initial viral rebound was observed at either day 7 or 14 (Fig. 4F), correlating with increased proviral DNA at these same time points. By linear regression analysis, the changes in plasma viral load (degree of viral rebound) significantly correlated with fold increase of proviral DNA in PBMCs at the same time points that viremia was initially detectable after an treatment interruption (*R* = 0.915; *P* = 0.0005), and roughly with absolute copies of proviral DNA (*R* = 0.42; *P* = 0.27) (Fig. 4G and H). These findings show a significant correlation between increased proviral DNA and viral RNA rebound and suggest that this is due to an increase in proviral DNA rather than total copies. More frequent sampling would be required to determine whether the rise in proviral DNA in cells predicts RNA viremia, but these data do have implications for viral pathogenesis and the source of a viral rebound in patients after treatment cessation.

## DISCUSSION

The duration of cART-free HIV remission after treatment interruption reflects the size of replication-competent viral reservoirs and determines whether treatment is highly effective (7, 52). However, reliable markers are desperately needed to assess persistent reservoirs under ART, predict viral rebound in treatment interruption, and improve strategies to prevent or diminish viral resurgence. In this study, we utilized the SIV-infected macaque model to (i) examine the effects of prolonged ART up to 20 months (considered long-term treatment in an animal model), (ii) longitudinally compare the degrees of decay of multiple cell-associated viral parameters under cART, and (iii) determine potential correlates or predictors of residual reservoir size (levels of integrated proviral DNA) with plasma viral rebound upon treatment interruption. The results showed that cART significantly reduced plasma and cell-associated SIV RNAs, yet both total viral DNA and integrated proviral DNA remained detectable in systemic and lymphoid compartments throughout cART. Interestingly, the dynamics of plasma viral load after viral rebound was very similar to those in primary SIV infection. Data indicated that the increase of proviral DNA in peripheral cells post-ART interruption, rather than total copies, correlated with plasma viral rebound at the timing of initially detectable plasma viral load once treatment was withdrawn. These findings suggest that increase of proviral DNA after treatment interruption may be useful for monitoring HIV persistence and reservoir size and to predict the recrudescence or magnitude of HIV. However, we could not detect this increase in proviral DNA in samples under cART.

In the viral life cycle, viral genetic mutation is introduced between the entry and integration steps. Genetic variation also occurs under selective pressures such as host immunity and therapeutic interventions and other natural factors, resulting in “clouds” of “quasispecies” (53, 54). However, more than 90% of proviruses in ART-treated patients might be replication defective due to internal deletions, mutations, premature stop codons, or defects in splicing and packaging signals (30, 55–57). Further, a proportion of intact proviral DNA may shift states from active transcription to a nonreactivable latent phase that persists in the host for a lifetime (58–60), propagating by cell division (61, 62) and even maintaining repressive silence when intact proviral DNA is integrated into the distinct “gene desert” sites of chromosomes, where integration sites are enriched in repressive chromatin marks (63). Due to these complexities, current scalable assays have been designed to measure viable viral reservoirs to better estimate the true size of the competent reservoir (31, 35, 50, 55, 58, 64–67), including quantitative viral outgrowth assays (QVOA) (25, 58, 68, 69), Tat/Rev-induced limiting dilution assays (TILDA) (45, 64, 65), and near-full-length individual proviral sequencing (FLIPS) methods (70, 71). Also, diverse proviral species may exist on distinct chromosomal sites in single cells from various tissues (estimated at <2% of lymphocytes in the blood) (39), and intact proviral DNA may produce recombinant/replication-incompetent virions at both the transcription and translation levels (55, 72–74), and/or by back mutation of defective proviral DNA, weakening the prediction of the results. Although sensitive, precise, and practical assays are well developed, there is still no real “gold standard” approach to accurately and reliably measure bona fide functional HIV/SIV reservoirs in cells, especially to detect residual replication-competent virus in both CD4^+^ T cells and myeloid cells (45, 65, 74–77). Biomarkers are urgently needed to predict viral persistence, effective therapy, and viral recrudescence. In this study, we combined a droplet digital PCR (ddPCR)-based US/MS viral RNA assay with nested quantitative PCR (qPCR) for proviral DNA quantification by primers targeting repetitive *Alu* DNA islands on macaque chromosomes and examined viral reservoirs in systemic and lymphoid tissues of SIV-infected macaques on long-term antiretroviral therapy to evaluate the viral replication and recrudescence after treatment interruption. Although the proviral DNA measured by qPCR is unable to distinguish the viral integration sites and intact/defective forms, the measurement of proviral DNA, quantified by chromosomal *Alu*-targeted nested PCR, still indirectly represents footprints of integrated provirus.

At day 7 post-cART interruption, PBMC-associated US SIV RNA and proviral DNA were detected in two of the macaques, but MS SIV RNA and circular 2-LTR were still undetectable, accompanied by essentially undetectable or very low viremia (Fig. 1 and 2). These results support the concept that HIV transcription may not necessarily lead to virus production because of the sequential HIV life cycle, possible defective proviral DNA, and/or posttranscriptional blocks at multiple levels (13, 64, 78–80). Ideally, a better predictor of viral rebound should be measured prior to ART interruption, so as to inform whether an HIV-infected patient could safely stop ART; fold-change of proviral DNA was thus evaluated under cART. Unfortunately, the results showed that proviral DNA in PBMCs essentially proceeded to decline in individual animals while on cART, which likely reflects the complicated dynamic interplay between contraction and expansion of cells containing provirus, leading to the observed slow decline in replication-competent reservoirs over many years. Besides viral DNA under cART, viral RNA forms measured prior to interruption could be expected to be associated with viral rebound. However, as indicated in Fig. 1, the levels of peripheral cell-associated SIV RNA (both US and MS RNAs) gradually declined after ∼2 months of cART treatment and progressed to undetectable (i.e., limiting analysis). Since detectable viral DNA residues under treatment are major sources to fuel viral rebound once analytic treatment interruption (ATI) is initiated, it is not unreasonable to expect a proportional relationship between levels of viral DNA and viral rebound after treatment interruption. The reason why cell-associated SIV RNA is not associated with proviral DNA under ART (i.e., proviral DNA positive but remaining viral RNA negative) might also include bystander activation-based expansions of cell clones, in which a large proportion of defective proviral DNA increases yet potentially fails to produce intact viral transcripts (81, 82). Compared with cell-associated US/MS SIV RNA, integrated SIV DNA rapidly increased as early as 1 week post-treatment interruption (Fig. 3E). Assuming that HIV-infected cells largely contain only one HIV DNA molecule in both peripheral blood and lymph nodes (46, 83, 84), increases in integrated proviral DNA likely reflect the turnover of cells harboring integrated proviral DNA, which is mostly driven by viral antigens, homeostasis, or proviral integration site-dependent proliferation (85–87).

Our data showed that US SIV RNA and plasma viral load were detectable at day 7 of ATI in some animals, which may be also detected earlier than this time point. Unfortunately, given sampling scheduling, we could not measure daily for the levels of these viral RNAs and counterpart proviral DNA during early time points after treatment interruption. However, the early/initial emergence of plasma viral load at the designated weekly schedule still essentially reflects the status of proviral DNA. The emergence of plasma viral load was differentially observed from weeks 1 to 3 in these SIVmac-infected animals after treatment interruption (Fig. 4H), probably attributable to multiple factors, such as virulence of SIVmac (versus HIV), ontogeny, genetics, reservoir size, clonal expansion, etc. (3, 71, 88). It is speculated that viral rebound preferentially resulted from large amounts of viral replication and new rounds of infection but not increases of proviral DNA. However, our data supported the concept that proviral DNA may be a correlate to ATI outcomes in SIV infection and short-term ART, as indicated by the following: (i) both plasma viral load and cell-associated SIV RNA were undetectable after ∼2 months of cART; (ii) only viral DNA was detectable throughout cART for either 6 or 20 months; (iii) increase of proviral DNA was detected in all animals once cART was withdrawn, to some extent; (iv) cell-associated SIV RNA and plasma viral load were detected off cART because of initiation of viral replication from proviral DNA; and (v) increase of proviral DNA significantly correlated with the emergence and magnitude of plasma viremia. All of these data are consistent with the converging consensus that a number of residues proviral DNA represent major obstacles for HIV eradication and cure. These data are also consistent with low proviral DNA levels showing delayed viremia rebound in HIV infection (22, 51). Recent advances highlight the HIV reservoir in tissue-resident myeloid cells in brain and other lymphoid tissues as sanctuaries of HIV persistence throughout ART (89–91). However, myeloid cells in blood and colon likely contain HIV transcripts but few proviruses in a large fraction of HIV^+^ patients under ART, compared with CD4^+^ T cells with readily detectable proviral DNA (92). Besides integrated proviral DNA, multiple viral rebound-associated factors, including tissue anti-HIV drug concentration, phylogenetic characteristics, and myeloid cell reservoir in tissues, were not measured in this study because of unavailable samples and will be investigated in future studies.

In summary, we report that increased proviral SIV DNA on ART is significantly correlated with the subsequent emergence and degree of plasma viral rebound after treatment interruption. There is also a subsequent increase in proviral DNA in PBMCs after ART interruption, even though it is expected that some of the proviral DNA might be transcriptionally silent at this early stage of treatment cessation (93). In SIV infection, the rapid increase of proviral DNA may significantly expand the reservoir size and restore viral reservoirs to pretreatment levels in systemic and lymphoid tissues after treatment interruption, which has implications for the use of nonhuman primates (NHPs) in evaluating treatment efficacy and informing treatment decisions.

## MATERIALS AND METHODS

### Ethics statement.

All animals in this study were housed at the Tulane National Primate Research Center in accordance with the Association for Assessment and Accreditation of Laboratory Animal Care International standards. All studies were reviewed and approved by the Tulane University Institutional Animal Care and Use Committee under protocol number P0305. Animal housing and studies were carried out in strict accordance with the recommendations in the *Guide for the Care and Use of Laboratory Animals* (94) (NIH, AAALAC number 000594) and with the recommendations of the Weatherall report *The Use of Nonhuman Primates in Research* (95). All clinical procedures were carried out under the direction of a laboratory animal veterinarian. All procedures were performed under anesthesia using ketamine, and all efforts were made to minimize stress, improve housing conditions, and provide enrichment opportunities (e.g., objects to manipulate in cage, varied food supplements, foraging and task-oriented feeding methods, and interaction with caregivers and research staff).

### Animals and virus.

Adult Indian-origin rhesus macaques (Macaca mulatta; RMs; *n* = 4) were intravenously inoculated with 100 50% tissue culture infective doses (TCID_50_) of SIVmac251. After 8 weeks, these animals received combined antiretroviral treatment (cART) with 3 anti-HIV drugs (tenofovir [TFV] at 20 mg/kg of body weight/day, emtricitabine [FTC] at 30 mg/kg/day, and dolutegravir [DTG] at 2.5 mg/kg/day) for 20 months. TFV and FTC were kindly provided by Gilead, Inc., and DTG was kindly provided by ViiV Healthcare. Blood and lymph node biopsy specimens were collected at different time points, and complete tissues at necropsy were analyzed as indicated (Fig. 1A). Whole blood was also collected before and after cART interruption from an additional 5 SIVmac251-infected macaques on 6-month cART, in which the viral strain, inoculation route, and antiretroviral drug combination were completely the same as for the 4 animals described above. Plasma and single-cell suspensions were prepared to examine plasma viral load and cell-associated viral DNA/RNA and for flow cytometry analysis.

### Tissue collection and phenotyping.

Fresh cells isolated from blood were stained and analyzed by flow cytometry as we previously reported (96). Cells were stained with CD3 (SP34), CD4 (OKT4; BioLegend), CD8 (SK1), and LIVE/DEAD fixable aqua dead cell stain kit (Invitrogen, Grand Island, NY). Isotype-matched controls were included in all experiments. All antibodies and reagents were purchased from BD Biosciences Pharmingen (San Diego, CA) unless otherwise noted. Samples were resuspended in BD stabilizing fixative (BD Biosciences) and acquired on an LSRFortessa (Becton, Dickinson, San Jose, CA). Data were analyzed with FlowJo software (Tree Star, Ashland, OR).

### Genomic DNA and total RNA extraction.

Fresh single cells, isolated from EDTA-treated venous blood by density gradient centrifugation with lymphocyte separation medium (MP Biomedicals, Santa Ana, CA) or lymph nodes at different time points, were processed to extract total genomic DNA and cellular RNA by the AllPrep DNA/RNA minikit (Qiagen) according to the manufacturer’s instructions. Viral RNA in plasma was directly isolated using the QIAamp viral RNA minikit (Qiagen). The extracted DNA and RNA samples were stored at –80°C until further processing.

### Quantification of plasma viral load and cell-associated SIV RNA transcripts.

The extracted RNA was reverse transcribed into cDNA using a SuperScript III first-strand synthesis system (Invitrogen) according to the manufacturer's protocol. Reverse transcription (RT) reactions were performed in a thermocycler at 25.0°C for 5 min and 50.0°C for 60 min, followed by an enzyme inactivation step at 70.0°C for 15 min. For the quantification of targets, all primer/probe sets were synthesized by Integrated DNA Technologies (IDT; Coralville, IA). Target hydrolysis probes contain on the 5′ end a fluorescence reporter dye, 6-carboxyfluorescein (FAM) or MAX (NHS ester), and on the 3′ end a quencher dye (Black Hole Quencher-1 or Iowa black FQ quencher [IBFQ]) as listed in Table S1. Plasma viral loads were measured by real-time PCR as we previously described (41). cDNA from cell-derived RNA was further used to quantify different SIV transcripts (unspliced SIV RNA targeting the conserved *gag* region and multiply spliced SIV RNA targeting the spliced SIV *tat*/*rev* junction) by digital droplet PCR (QX100 droplet digital qPCR system; Bio-Rad) with minor modification as previously published (64). Samples were run in duplicate in a 20-μl volume containing Supermix, 250 nM primers, 900 nM probe, and 2 μl of undiluted cDNA under the following cycling conditions: 10 min at 95°C and 40 cycles of 94°C for 30 s and 63°C for 60 s, followed by final 98°C for 10 min. Droplets were analyzed by QuantaSoft software in the absolute quantification mode. Copies of SIV transcripts expressed as copies per one million cells (PBMCs or/and mononuclear cells in the lymph node) were measured and normalized to cellular input, as determined by copies of genomic CCR5 (single-copy rhesus macaque CCR5 DNA per cell) (5, 23, 97–99). The limit of detection (LOD) was based on three or more replicates and calculated using GenEx 5 (MultiD Analyses AB).

### Quantification of cell-associated SIV DNAs.

To ensure that quantifications of total SIV DNA, 2-LTR DNA, and integrated proviral DNA were comparable, a series of specific standards (plasmids containing SIV U5 DNA and 2-LTR DNA junction) were prepared to perform nested PCR. Since HIV preferentially integrates into regions of the chromosome on which repetitive *Alu* DNA islands are widely dispersed and in close proximity to the integrated proviral DNA (100), two *Alu* primers were used to amplify the segments of integrated proviral DNA, in combination with the primer of the LTR U5 region. In comparison, qPCR with primer pairs targeting the LTR U5 fragment only was used to measure total SIV DNA, including a proportion of integrated proviral DNA. Two-step PCR amplification was run in parallel to quantify total viral DNA as described previously (101–104). Briefly, the preamplification reactions in triplicate tubes per sample were performed using SIV long terminal repeat primer and two outward *Alu* primers (integrated SIV DNA), or primer pairs of U5 (total SIV DNA) and 2-LTR (circular SIV DNA), on 7900HT sequence detectors (Life Technologies). The reactions were performed as follows: 25 μl of the reaction mix, containing 1× PCR buffer, 0.2 mM deoxynucleoside triphosphates (dNTPs), 2 mM MgCl_2_, 0.8 μM each primer, and 0.5 U of *Taq* DNA polymerase (Invitrogen Life Technologies), was programmed to perform a 5-min hot start at 95°C, followed by 20 cycles of denaturation at 95°C for 30 s, annealing at 63°C for 30 s, and extension at 72°C for 3 min. A total of 2.5 μl of these amplicons were further amplified in triplicate with each primer/probe pairs (Table S1) by real-time PCR using 40 cycles at 95°C for 15 s and 63°C for 1 min. The highly reproducible calibration curves were generated by plotting quantification cycle (*C_q_*) values against log-transformed concentrations of the serial standard. Internal standard curves were also generated using a known copy number of target plasmids (1 to 500 copies) diluted in cellular DNA from SIV-naive RMs. The calibration curves and the internal regression curves were used for interpolating initial copies of each target in unknown samples. A nontemplate control (NTC) and extracted cellular DNA from the HUT78/SIVmac239 cell line (positive control) were included in the qPCRs. As described above, quantification of SIV RNA/DNA was expressed as copies per 1 million cells, in which cell numbers were determined by copies of genomic CCR5 DNA per cell. To avoid individual discrepancy of host immunity, we also calculated and analyzed the degree of decreased or increased SIV RNA/DNA, including the ratio of longitudinal plasma viral load and cell-associated SIV after treatment interruption to the baseline at the time of preinfection or/and final cART treatment, expressed as relative changes or fold increase.

### Statistical analysis.

Descriptive analyses, including data visualization and plot generation, were accomplished mainly using GraphPad Prism 4.0 (GraphPad Software, San Diego, CA). Statistical comparison between groups at different time points was achieved using two-tailed paired *t* tests or Wilcoxon matched-pairs signed-rank test (GraphPad Software). A nominal α level of 0.05 was used to define statistical significance. The relationship was analyzed using a linear regression model as implemented in R’s lm function. The regression was made using the ggpubr R package (https://rpkgs.datanovia.com/ggpubr/).

## Supplementary Material

Supplemental file 1
