# Supplementary material for: Increased Proviral DNA in Circulating Cells Correlates with Plasma Viral Rebound in Simian Immunodeficiency Virus-Infected Rhesus Macaques after Antiretroviral Therapy Interruption
Source: J Virol. 2021 Feb 24;95(6):e02064-20. doi: 10.1128/JVI.02064-20 (PMC8094949; doi:10.1128/JVI.02064-20)
Supplement: Supplemental file 1 [file JVI.02064-20-s0001.pdf]

Supplementary Table 1. Primers and hydrolysis probes sequences for targets.

Target	Oligonucleotide	Sequence ('5 to 3')	Amplicon length
**Rh CCR5**	Forward primer	CCAGAAGAGCTGCGACATCC	
**DNA**	Reverse primer	CTAATAGGCCAAGCAGCTGAGG	75bp
	Probe	(MAX)-TTCCCCTAC/ZEN/AAGAAACTCTCCCCGGTAAGTA-(IBFQ)	
**SIV LTR U5**	Forward primer	AGGCTGGCAGATTGAGCCCTGGGAGGTTC	
**DNA**	Reverse primer	CCAGGCGGCGACTAGGAGAGATGGGAACAC	200bp
	Probe	(FAM)-TTCCCTGCT/ZEN/AGACTCTCACCAGCACTTGG-(BHQ-1)	
**Circular 2-LTR**	Forward primer	TAAGCTAGTGTGTGTTCCCAT	
**DNA**	Reverse primer	CTCCTGTGCCTCATCTGATACA	305bp
	Probe	(FAM)-AGTCGCCGC/ZEN/CTGGTCAACTCG-(BHQ-1)	
**Alu DNA**	Reverse primer-1	GGCGCCGGAGGGTTTCAC	
	Reverse primer-2	GAACTCCTGAATTCAAGTGATTGTCC	
**Unspliced SIV**	Forward primer	GTCTGCGTCATCTGGTGCATTC	
**RNA**	Reverse primer	CACTAGGTGTCTCTGCACTATCTGTTTTG	84bp
	Probe	(FAM)-CTTCCTCAG/ZEN/TGTGTTTCACTTTCTCTTCTGCG-(BHQ-1)	
**Multiply spliced**	Forward primer	TGAGCAGTCACGAAAGAGAAG	
**SIV RNA**	Reverse primer	GTATCAGTTGGCGGATCAGG	202bp
	Probe	(FAM)-AAGGAGACG/ZEN/GTGGAGAAGGCG-(BHQ-1)	
